# Prevalence and short-term outcome of hepatorenal syndrome: A 9-year experience in a high-complexity hospital in Colombia

**DOI:** 10.1371/journal.pone.0239834

**Published:** 2020-10-20

**Authors:** Margarita Rey R., Andrés F. Delgado, Alejandra De Zubiria, Renzo Pinto, José A. De la Hoz-Valle, Erika D. Pérez-Riveros, Gerardo Ardila, Fernando Sierra-Arango

**Affiliations:** 1 Gastroenterology and Digestive Endoscopy Section, Fundación Santa Fe de Bogotá, Bogota, Colombia; 2 Internal Medicine Department, Fundación Santa Fe de Bogotá, Bogota, Colombia; 3 School of Medicine, Universidad de Los Andes, Fundación Santa Fe de Bogotá, Bogota, Colombia; 4 Transplant Service Department, Fundación Santa Fe de Bogotá, Bogota, Colombia; 5 Clinical Studies and Clinical Epidemiology Division, Fundación Santa Fe de Bogotá, Bogota, Colombia; Memorial University of Newfoundland, CANADA

## Abstract

**Background & aims:**

Hepatorenal syndrome is a rare entity that is part of the complications of liver cirrhosis in its more severe stages. Without treatment, its mortality rate increases significantly. Terlipressin is considered to be the therapy of choice until the need of a liver transplant. The aim is to determine its prevalence, define patients’ characteristics, triggers and 90-day survival, according to the type of managements established.

**Method:**

This was a retrospective cohort study conducted in Colombia. It included patients with cirrhosis and acute kidney injury who met hepatorenal syndrome criteria, reaching 28 patients from 2007 to 2015. Groups were categorized according the type of hepatorenal syndrome and treatment. Demographic and trigger factors were evaluated to characterize the population. Treatment outcomes with terlipressin vs norepinephrine were analyzed up to a 90-day survival, using log Rank test. Continuous variables needed Student's T and Mann Whitney's U tests and categorical variables, Chi2 test. A value of p <0.05 and a power of 85% was considered. The data was analyzed in the SPSS version 23 software.

**Results:**

117 patients with cirrhosis developed renal injury; of these 23.9% were diagnosed with Hepatorenal Syndrome (67.8% type1; 32.1% type2). The presence of ascites was 100% in HRS2 and 84% in HRS1 (p = 0.296). The main trigger in both types was paracentesis greater than 5 liters in the last 4 weeks (39.3%). In total, 35% of the patients received renal replacement therapy and 14% underwent a hepatic transplant. Type 1 was more frequent (63% received terlipressin; 21% norepinephrine). The total complete response was 36% (Type2 66.6% vs. Type1 18.7%) (p = 0.026). In contrast, the overall mortality was of 67.8% at 90-day of follow-up (89.4% Type1 vs. 22% Type2) (p = <0.001). We found a lower mortality rate in patients treated with terlipressin than treated with norepinephrine (p = 0.006).

**Conclusion:**

There is scarce clinical and epidemiological information about this condition in Colombia. A significant difference between the two drugs cannot be stipulated due to the limitation in the sample size of our study. The general mortality at a 90-day follow-up was high, being higher in patients with HRS1. While the results of this study are suggestive of clinical information for HRS patients in the Colombian population, they should also be interpreted with caution, therefore further multicenter studies should be performed.

## Introduction

Hepatorenal syndrome (HRS) is a rare entity managed primarily in high complexity institutions. It is part of the complications of cirrhosis and advanced liver failure [1]. This syndrome is characterized by a severe renal failure in cirrhotic patients caused by splanchnic vasodilatation, decreasing the effective circulating volume; this produces renal vasoconstriction, reducing glomerular filtration [2]. Its reported prevalence is variable, (7–45%) [3–5]. HRS is classified in two main clinical forms: HRS type 1 and HRS type 2 [6]. These differ in the progression and severity of the symptoms, HRS1 being more severe and of rapid progression [7]. This condition is considered the most frequent fatal complication in patients with cirrhosis. Without treatment, it has a 2-week high mortality rate (80% in patients with HRS1) [8]; a total 3-month survival of 10% [9].

There have been many updates of the International Club of Ascites (ICA) criteria for HRS. Most recently in 2015 Angeli et al. [10], reported evidence supporting this last proposal of approach to the diagnosis and treatment of this condition. Some HRS triggers described in medical literature are: episode of bacterial infection (spontaneous bacterial peritonitis is the most common) [11,12], intravascular volume contraction due to bleeding [13], excessive use of diuretics [14], large volume paracentesis [15], major surgery or acute liver failure [16]. These are more frequently associated with the development of type 1 HRS rather than HRS type 2 [9].

The understanding of the pathophysiological mechanisms that produce the HRS, allows the introduction of pharmacological interventions as bridge therapy until the moment of liver transplant [17]. This has increased short-term survival [9]. Currently, terlipressin, a vasopressin analog vasoconstrictor, in combination with albumin is the bridge therapy of choice for patients with type 1 HRS [18]; reducing mortality and improving renal function of patients with this condition [19]. Rodriguez et al. [20], mentions that terlipressin is generally well tolerated and reverses HRS2, despite its common relapse. However, low evidence of long-term efficacy and safety has been demonstrated in patients with HRS2 [21]. The prognosis with any drug therapy remains poor and liver transplant is the definitive treatment [22]. Some benefits are described in the use of terlipressin in pre-transplant and post-transplant period, decreasing morbidity and improving patient outcomes [23].

There is scarce clinical and epidemiological information about this condition in Colombia. It could be questionable to combine data from patients with HRS1 and HRS2, given their different course and treatment response. However, given the lack of information, this study seeks to cover aspects of both types of HRS. The objective of this study is to determine the prevalence of the disease, describe the characteristics of the patients with HRS and the triggers, as well as the 90-day survival, according to the type of management established at a fourth level hospital.

## Materials and methods

### Patients

The cohort included hospitalized patients at Fundación Santa Fe de Bogotá, with a diagnosis of HRS between January 2007 and December 2015. The study was approved by our institutional ethics committee; It was done and followed according to the Declaration of Helsinki and the Resolution 8430, 1993 [24,25].

Despite the recent updates of the ICA criteria for HRS, this study is based in 2007 ICA diagnostic criteria, given the years of data recruitment. This criteria was based on the exclusion of other causes of acute kidney injury and assessing the response to volume expansion for 48 hours [26]: 1. liver cirrhosis and 2. Creatinine ≥1.5 mg/dL on any day of hospitalization [19,27]. Additionally, hospitalized patients over 18 years old with clinical, biochemical, imaging, endoscopic or cirrhosis biopsy diagnose were included. As well as patients with creatinine persistence ≥1.5 mg/dL after 48 hours of suspending diuretics and volume expansion with albumin 1 gr/kg/day (Maximum 100 gr/day). Those patients with sepsis, shock of any etiology, heart failure, respiratory failure, coronary heart disease, stroke, renal disease (proteinuria> 500 mg/day, more than 50 red blood cells per field of high power or obstructive nephropathy), hepatocellular carcinoma, use of nephrotoxic drugs, renal abnormality visualized by ultrasound and patients with incomplete clinical history were excluded from the study.

Patients were categorized into HRS type 1: patients with twice the initial creatinine (greater than 2.5 mg/dL) in less than 2 weeks; HRS type 2: creatinine ≥ 1.5 mg/dL and with slow progressive course. Demographic factors were evaluated: age, sex, the etiology of cirrhosis; clinical variables: heart rate, blood pressure, urinary output, temperature, presence of ascites, presence of hepatic encephalopathy; and laboratory variables (leukocyte count, hemoglobin, platelet count, sodium, potassium, bilirubin, INR, albumin and creatinine measured daily). Taking into account these variables, Child-Pugh and MELD were calculated from the laboratory results when diagnosing kidney injury.

### Risk factor

The frequency of risk factors for the development of HRS 1 or 2 were identified as: spontaneous bacterial peritonitis defined as ≥250 polymorphonuclear in ascites fluid [12]; as well as bacterial infection in the last week [11], paracentesis greater than 5 liters in the last 4 weeks [15], use of non-steroidal anti-inflammatory drugs (NSAIDs) in the previous 2 weeks, use of nephrotoxic medications in the previous 2 weeks and upper gastrointestinal bleeding in the last 6 weeks [13].

### Outcomes

The treatment outcomes with terlipressin and norepinephrine were analyzed, as well as the dosage of each medication in patients with HRS1 and HRS2. In order to avoid bias in the evaluation of terlipressin and norepinephrine in both HRS1 and HRS2, subgroups of analysis were performed as follows: Group 1: HRS1 + Terlipressin; Group 2: HRS1 + Norepinephrine; Group 3: HRS2 + terlipressin; Group 4: HRS2 + norepinephrine. The treatment with terlipressin was administered according to the medical criteria supported in those referenced by Salerno et al [28] as soon as this was approved by the National Institute of Food and Drug Surveillance of Colombia (INVIMA for its acronym in Spanish) for this disease in 2011 [29]. Prior to that, all cases were managed with norepinephrine influenced by the hemodynamic condition of the patient.

As mentioned above, patients who were treated with albumin prior to this study were included, and after the onset of vasopressor, albumin was continued at 1g / kg / day while the patient was on vasopressor infusion; it was only suspended at the moment the patient finished the therapy. Additionally, it is important to clarify that all patients were admitted into the ICU according to the institution's protocol, given the type of drugs that were administered to the patients. Norepinephrine is administered via the central line and terlipressin could be administered by either central line or peripheral IV. This is why all our patients were managed in the ICU. Complete HRS resolution outcomes were defined as a decrease of creatinine less than 1.5 mg/dL. This study estimated the resolution time according to therapy [19], and a 90-day mortality according to the type of HRS and treatment received.

### Statistical analysis

A descriptive analysis of the variables was performed according to the HRS type. Continuous variables were described with measures of central tendency and dispersion (mean and standard deviation) and categorical variables are described using proportions. Overall survival was described according to the type of HRS and according to the type of treatment administered with Kaplan Meier survival curves.

Subsequently, the presence of statistical associations between the different clinical variables and the type of HRS were evaluated. For the continuous variables, the Student's T and Mann Whitney's U tests were used and for the categorical variables the Chi2 test. Survival curves were compared using the log Rank test. A value of p <0.05 was considered to find a significant statistical difference. The data was analyzed in the SPSS version 23 software.

The sample size was not calculated a priori, it was performed with post hoc analysis with the variables of interest finding a power of 85% to find a difference between the mortality of the different types of HRS. All patients that met the inclusion and exclusion criteria were included.

## Results

During the 9-years of study, 117 patients with cirrhosis who developed kidney injury were hospitalized. The diagnosis of HRS was made in 23.9% (n = 28); 67.8% were HRS1 and 32.1% HRS2. It is important to clarify that 3 patients were excluded from the survival analysis. One of these patients was not managed with any vasoactive agent, as palliative management was considered. The remaining two were handled with double vasoactive agents as follows: norepinephrine 0.16 mcg/k/min + Terlipressin 1 mg every 4h. This patient resolved the HRS on day 7, however, he died on day 41 of follow-up. The second patient received: Norepinephrine 0.06 mcg/k/min + Terlipressin 1 mg every 6h. This patient died on day 3 was followed-up.

### Patient characteristics

Type 1 HRS was more frequent in male patients, who presented a greater compromise of liver and renal function. The average age for HRS1 was 55.5 years (SD = 13.1) and 59 years (p = 0.35) in HRS2 (SD = 5.76). HRS2 was more frequent in women (66.6%). Regarding the etiology of cirrhosis, the first cause was alcoholic (42.1%) for HRS1 and for HRS2 it was non-alcoholic steatohepatitis (66.6%). (Table 1).

**Table 1:** Demographic of the study population (n = 28).

VARIABLE	HRS TYPE1 (N = 19)	HRS TYPE 2 (N = 9)	P-VALUE
Age (years) (SD)	56 ± 13.1	59 ± 5.76	0.35
Women	36.8%	66.6%	-
Alcoholic Hepatopathy	42.1%	11.1%	-
Viral Hepatopathy	0%	11.1%	-
Alcoholic + viral	0%	0%	-
Autoimmune	15.8%	0%	-
Non-alcoholic steatohepatitis	15.8%	66.6%	-
Other	26.3%	11.1%	-
CHILD-PUGH B	26.3%	55.5%	0.999
CHILD-PUGH C	73.7%	44.5%	0.02
MELD average (SD)	32.1 ± 7.6	21.5 ± 3.4	<0.001
Heart Rate (BPM) (SD)	71.8 ± 14.1	71.1 ± 9.8	0.88
Temperature (centigrade) (SD)	36.4 ± 0.5	36.7 ± 0.6	0.25
Urinary Output (cc/k/h) (SD)	0.6 ± 0.6	0.54 ± 0.3	0.71
Systolic Blood Pressure (mmHg) (SD)	99.7 ± 13.7	98.1 ± 16.1	0.79
Diastolic Blood Pressure (mmHg) (SD)	57.3 ± 10.7	57.3 ± 9.6	0.99
Ascites	84.2%	100%	0.296
Hepatic encephalopathy (Some degree)	52.6%	11.1%	0.007
Leukocytes (cell/mm^3^)	7698 ± 3575.8	6577 ± 2593.6	0.36
Hemoglobin (g/dL)	10.4 ± 2.3	10.8 ± 1.4	0.62
Platelets (cell/mm^3^)	81842 ± 45397.3	129777 ± 62786.9	0.06
Serum creatinine (mg/dL)	3.0 ± 1.0	2.6 ± 2.0	0.046
Total bilirubin (mg/dL)	22.1 ± 15.9	1.9 ± 1.1	<0.001
Potassium (meq/L)	4.2 ± 1.2	5 ± 0.9	0.06
Serum Sodium (meq/L)	131.7 ± 6.4	130.3 ± 5.0	0.54
Albumin (g/L)	2.3 ± 0.8	2.8 ± 0.8	0.20
International Normalized Ratio (INR)	1.9 ± 0.7	1.3 ± 0.2	0.003

SD: Standard Deviation.

BPM: Beats per minute.

Regarding the severity of liver disease, 73.7% of patients with HRS1 had Child C cirrhosis (p = 0.02), while in HRS2 the proportion between Child B and C was almost similar (55.5% and 44.5% respectively). Patients with HRS1 had a higher average of MELD score than patients with HRS2, (p = <0.001). There were no differences in the physiological variables in both types of HRS (heart rate, respiratory rate, blood pressure, temperature and urinary expenditure). The presence of ascites was of 100% in HRS2 and 84% in HRS1 (p = 0.296). Hepatic encephalopathy occurred 5 times more in HRS1 than in HRS2 (52% and 11%) with a p = 0.007. (Table 1).

According to the laboratory results, the total leukocyte count had no differences, as was hemoglobin, although the platelet count was lower in HRS1, but without statistical significance. In liver profile tests, bilirubin was considerably higher in HRS1 with an average of 22.1 mg/dL compared to 1.9 mg/dL in HRS2, with a significant statistical difference. The INR also showed a greater alteration in HRS1 than in HRS2 (1.9 and 1.3 respectively) p = 0.003. (Table 1).

### HRS triggers

The main trigger of HRS in both types was paracentesis greater than 5 liters in the last 4 weeks (39.3%), followed by an unknown cause (32.1%) and infection from another site in the last week (17.9%). The second cause in HRS1 was of unknown origin (28.6%) and in HRS2 it was the infection of another area in the last week (7.1%). Among the triggers, the use of NSAIDs or nephrotoxic in the last two weeks was not found (Table 2).

**Table 2:** HRS triggers.

Triggers	Type 1 n = 19	Type 2 n = 9	TOTAL n = 28
Paracentesis greater than 5 liters in the last 4 weeks	17.9%	21.4%	39.3%
Infection from another site in the last week	10.7%	7.1%	17.9%
Intestinal bleeding in the last 6 weeks.	7.1%	0%	7.1%
Spontaneous Bacteria Peritonitis	3.6%	0%	3.6%
Use of NSAIDs in last 2 weeks	0%	0%	0%
Use of nephrotoxic drugs in the last two weeks	0%	0%	0%
Unknown cause	28.6%	3.6%	32.1%

### HRS treatment

In HRS1 63% received terlipressin, while 21% received norepinephrine. HRS2 had similar findings, 66.6% was treated with terlipressin and 33.3% with norepinephrine. There was a small group of HRS1 patients who were treated with the combination of terlipressin plus norepinephrine and represented 10.5% (2/19) of the study. On the other hand, a HRS1 patient did not receive any treatment, since palliative management was defined.

The average dose of terlipressin used in HRS1 was 8.8 mg and for HRS2 it was 6.2 mg. The average dose of norepinephrine used in HRS1 was 0.45 mcg/k/min and for HRS2 it was 0.40 mcg/k/min (Table 3).

**Table 3:** HRS treatment.

VARIABLE	HRS TYPE 1 (n = 19)	HRS TYPE 2 (n = 9)	p-Value
Terlipressin	12/19 (63.1%)	6/9 (66.6%)	-
Average dose of terlipressin	8.8 mg (DS 3.5)	6.2 mg (DS 3.1)	0.18
Norepinephrine	4/19 (21%)	3/9 (33.3%)	
Average dose of norepinephrine	0.45 mcg/k/min (DS 0.13)	0.40 mcg/k/min (DS 0.26)	0.86
Terlipressin + Norepinephrine	2/19 (10.5%)	0	-

### Other HRS treatments

Of the 28 patients with HRS, 35% required renal replacement therapy with hemodialysis, of which 70% had a diagnosis of SHR1. Of all dialyzed patients, 90% died within 90 days and 10% received a liver transplant as definitive therapy. In our cohort, liver transplantation was accomplished in 4 of the 28 patients, the procedure was performed 3-13-38-153 days after the diagnosis of HRS, respectively, achieving a survival of more than 90 days in 3 patients.

### HRS resolution

The total complete response defined as creatinine decrease of < 1.5 mg / dL, was 36%. HRS2 resolved more than HRS1 (66.6% vs. 18.7%), regardless of the vasopressor. Differentiating by subgroups: HRS1 treated with terlipressin, it resolved the HRS in 2/12 (16.6%), unlike those treated with norepinephrine which were 1/4 (25%). On the other hand, the HRS2 patients that received terlipressin, 3/6 (50%) resolved and with norepinephrine 3/3 (100%) (Table 4).

**Table 4:** Complete HRS resolution according to the treatment.

	Complete HRS resolution		Average time (days) of HRS resolution		
**Variable**	HRS1 (n = 16*)	**HRS2 (n = 9)**	**HRS1**	**HRS2**	**p-value**
Terlipressin	2/12 (16.6%)	3/6 (50%)	4.5 ± 2.12	6 ± 1.73	0.55
Norepinephrine	1/4 (25%)	3/3 (100%)	5 ± N/A	5 ± 1	1.0
Any Vasopressor support	3/16 (18.7%)	6/9 (66.6%)	5.25 ± 1.71	5.5 ± 1.38	0.80

* Two HRS1 patients received terlipressin plus norepinephrine and one patient did not receive any treatment were excluded.

**Fig 1:**
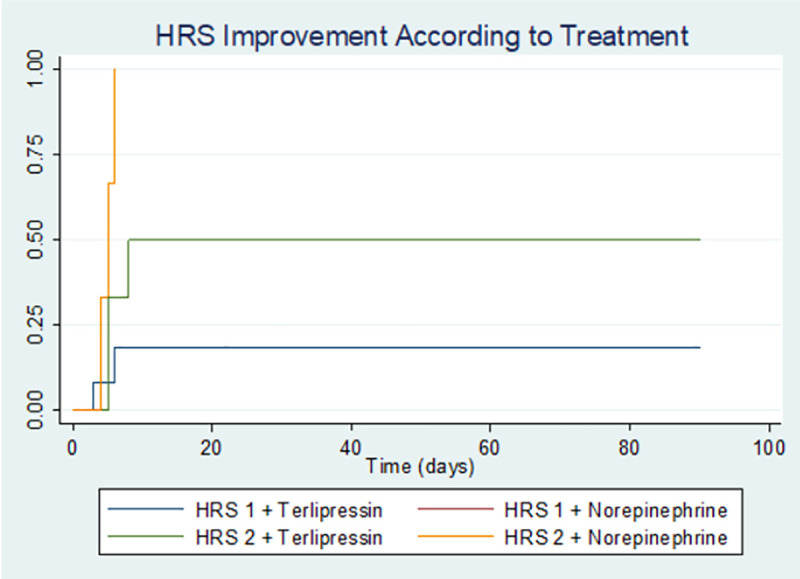
HRS resolution according to vasopressor support. P value = 0.026.

The average time of general resolution was of 5.25 days in type 1 and 5.5 in type 2 (p = 0.8). In HRS1 the resolution time with terlipressin was of 4.5 days and in HRS2 it was of 6 days. In patients treated with norepinephrine, the resolution time was of 5 days for both, patients with HRS1 and HRS2 (Table 4). There was a significant statistical difference in the complete resolution of HRS1 and HRS2 according to the different treatments (p = 0.026). Additionally, patients who did not recover in the first 10 days, were less likely to improve later. (Fig 1).

### Mortality

**Fig 2:**
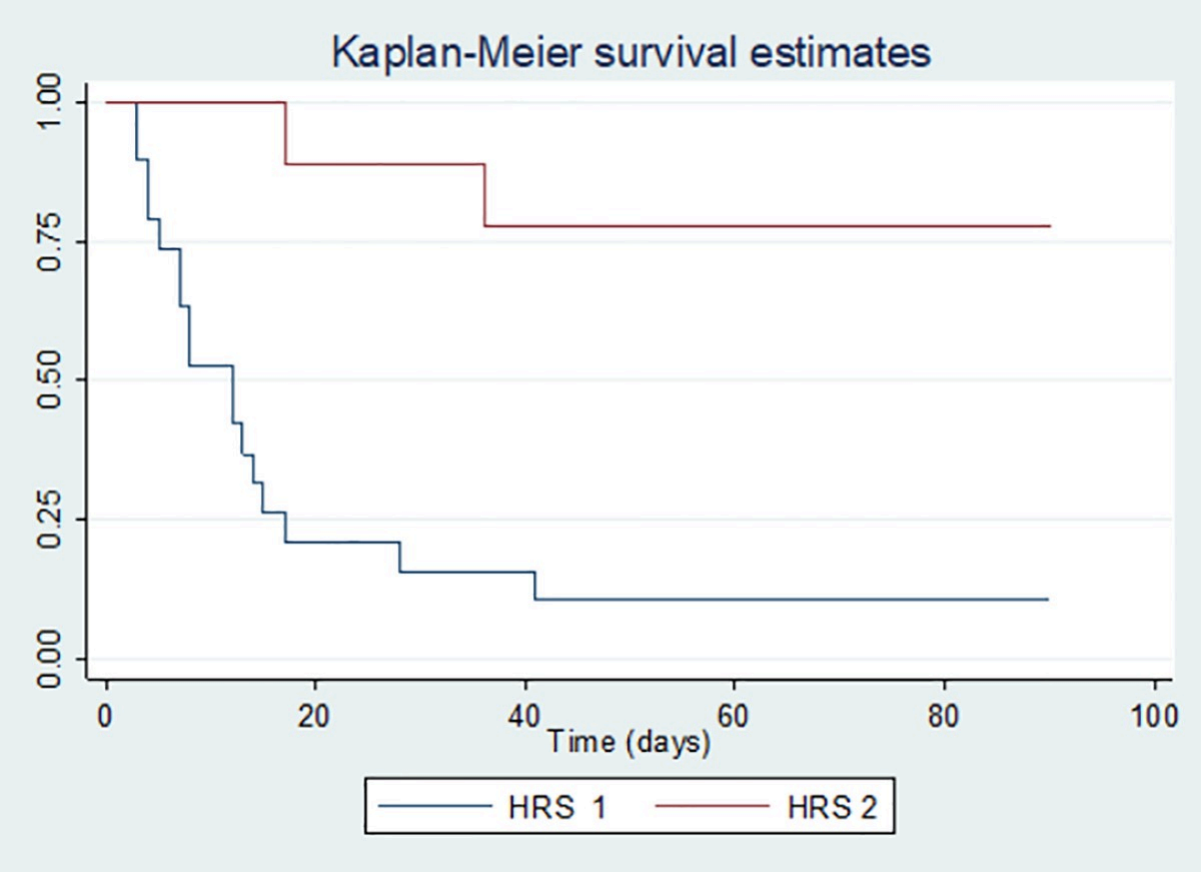
HRS mortality (Kaplan Meier Curve). P value = <0.001.

**Fig 3:**
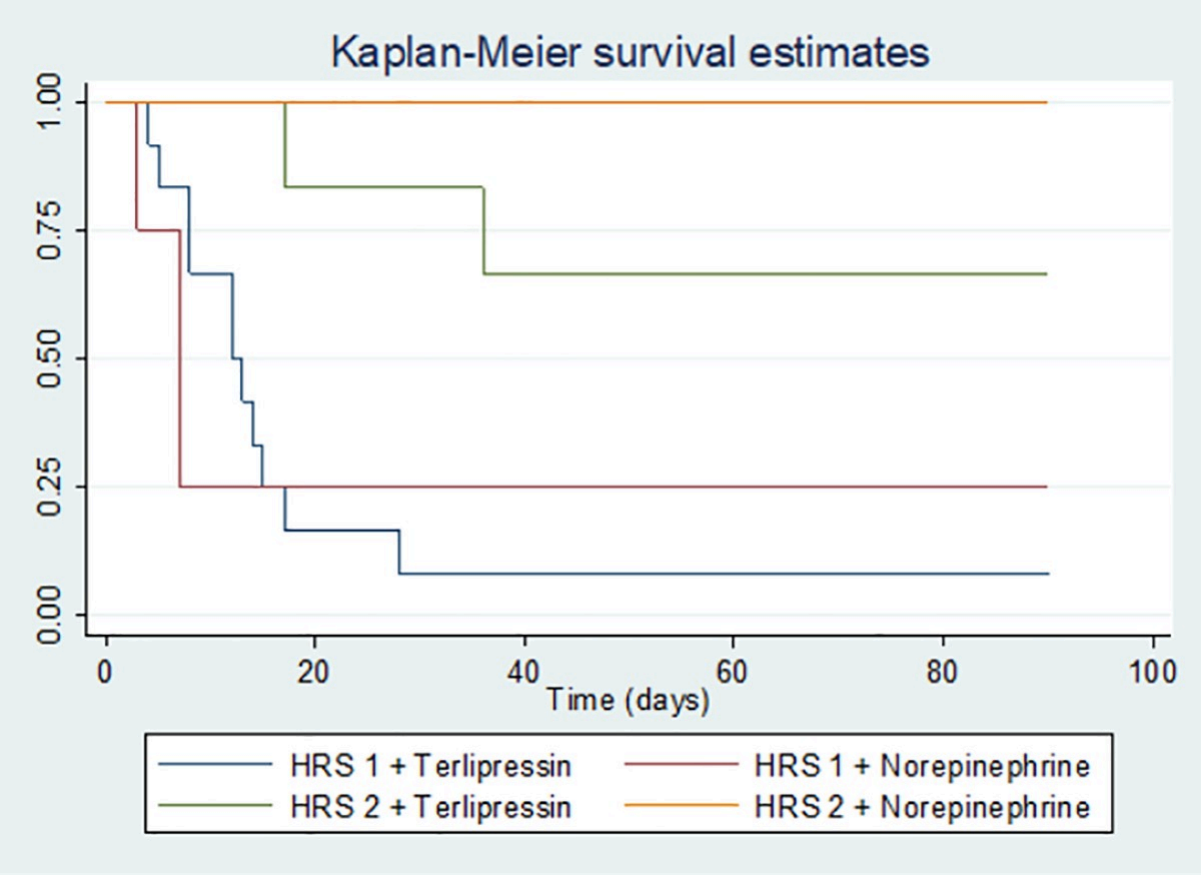
HRS mortality according to treatment (Kaplan Meier curve). P value = 0.006.

Of the total number of patients, 19 patients died, corresponding to a mortality of 67.8% at a 90-day follow-up (HRS1 89.4% and 22% in HRS2) (p = <0.001) (Fig 2). Of the total number of patients who responded to drug therapy (9/28), three of them died within 8-17-41 days respectively, all with a diagnosis of HRS1. On the other hand, those who did not respond had a mortality of 94% at 3 months; finding a significant statistical difference in the survival rate of HRS according to the different treatments. This showed a lower mortality rate in patients treated with terlipressin than with norepinephrine (p = 0.006). (Fig 3).

## Discussion

Patients with advanced liver cirrhosis have a higher risk of complications affecting their life expectancy [5]. Kidney injury is one of the most frequent complications, especially when portal hypertension is associated [30]. HRS is the ending result of sustained reduction of renal perfusion in the context of advanced liver disease; it is associated with a poor prognosis [31].

The prevalence of HRS observed in this study was of 23.9% (67% HRS1 and 33% HRS2). HRS was the cause for acute kidney injury in one out of every 4 patients. Several studies determine the prevalence of HRS, finding a significant variation depending on the HRS definition used and the inclusion and exclusion criteria considered. Recently, Seetlani et al [32] reported that 15% out of 265 patients with cirrhosis were diagnosed with HRS. However, Salerno et al [19] reported that, out of 253 patients with cirrhosis and kidney injury, 45.8% had HRS (30% HRS1 and 15.8% HRS2). In which case the prevalence is higher in HRS1, just like in this study. In Colombia there are no studies so far; reason why this study was based on the 2007 ICA criteria [28].

Regarding the characteristics of our population, we found that the average age of presentation had no significant difference between both types of HRS. HRS1 appeared more frequently in men while HRS2 appeared more in women. In the study by Licata et al. [33] a significant relationship similar to this study was observed. On the other hand, the majority of patients with HRS1 had Child C liver cirrhosis and a higher MELD score. Additionally, these patients presented a high INR, decreased platelet count and a greater presence of hepatic encephalopathy compared to HRS2. These findings are related to the severity of liver involvement and are similar to those reported in literature [19,33,34].

The main cause of cirrhosis in type 1 HRS was alcoholism while in type 2 it was non-alcoholic steatohepatitis. In literature, alcoholic cirrhosis is observed as one of the most frequent causes without discriminating the type of HRS [1,19]. For both types of HRS the most frequent trigger was paracentesis greater than 5 liters in the last 4 weeks. It is important to clarify these patients received the albumin replacement treatment for the paracentesis procedure. This is different from what was reported in other studies, where spontaneous bacterial peritonitis is observed as the most frequent trigger [14,35,36]. It is noteworthy that unlike the literature, in our population, spontaneous bacterial peritonitis was not the most frequent trigger. Knowing the low frequency of this condition in our community, in Latin America, countries like Peru showed that 20% presented this as a trigger, a percentage similar to that of this study [37].

The standard treatment for HRS1 patients is vasopressor agents. In numerous studies vasopressors have been used in HRS2 [38]; two metanalysis showed no significant difference between the use of norepinephrine and terlipressin, nor among the types of HRS [8]. Although, adverse events were less common with norepinephrine (OR = 0.36, 95% CI 0.15 to 0.83; p = 0.017) [39]. However, there is insufficient evidence to recommend this in HRS2 and it will depend on the physicians’ criteria.

In this case, one of the patients with HRS1 had palliative care and other two patients used two vasopressor agents (excluding them from the analysis), generating a higher percentage of HRS2 patients with treatment. Salerno et al [19], found similar results with 84% and 72% vasopressor interventions, respectively. The medication dosage used in the treatment of HRS was higher for HRS1 compared to HRS2, but there was no significant difference with the dosage between the two medications.

In this study, the complete response to the treatment was of 36%. The results at a global level correspond to a value greater than 50%. In other randomized studies in Europe in 2015 and 2016 the resolution was from 19% to 55% [40,41]. This difference in data from this work in relation to other studies could be related to the use of other vasopressors such as midodride/octreotide and the variability of the studied patients at the demographic level [42]. In a recent meta-analysis, Jiang et al [43]. collected 9 eligible articles from observational studies of HRS patients, determining an estimated average response to vasopressor of 32%, which is comparable with the results of our study.

The complete resolution focusing only on the type of HRS, resolves better in patients with HRS2 (66%) vs HRS1 (21.7%). This data is similar to those found in literature given a greater compromise of liver function in patients with HRS1 [44]. Another reason is, the prognosis is dependent on the severity of kidney injury. This suggests that renal compromise in HRS1 decreases the probability of response to any vasoactive treatment [42,45]. In patients with HRS1, the non-response to terlipressin was of 83.4%. This was higher than global studies where an average of 54% did not respond to vasopressor management [27]. Thus, in this study the number of patients with terlipressin was higher than those with norepinephrine.

It is important to highlight the full resolution (100%) in patients with HRS2 treated with norepinephrine. However, this can be explained given the low number of patients treated in this group. When comparing norepinephrine with terlipressin in other studies, they show similar efficacy [27]. There is no data on dual management with two vasopressors, as this can generate synergy and increase the likelihood of adverse effects. In this study, two patients who started management with terlipressin required additional management with norepinephrine due to the presentation of hypotension.

A high overall HRS mortality was found (67%); with a higher mortality of HRS1 in relation to HRS2, 89% and 33% respectively. This is described in multiple studies; HRS1 patients have a higher mortality rate than patients with HRS2 [8,10,46]. This indicates a greater complication of cirrhosis in patients with HRS1.

Therefore, survival was greater in patients with HRS2. Of which, those who responded adequately to vasoactive management obtained a survival of 70% at 3 months, however, half of these patients underwent a hepatic transplant, reason why we think this could influence the survival of the patients. While patients who did not respond to vasopressor treatment, survival decreased significantly to 5.6%. In this study, it was difficult to establish differences in mortality according to the vasopressor support given that the sample size was small. Despite this, terlipressin was significantly superior to norepinephrine. However, there is a Cochrane review that found no difference in mortality when comparing terlipressin to norepinephrine [47].

The sample size of this study could be a limitation despite the prolonged time in which the sample was taken. Additionally, this was a retrospective study, where there is a point of improvement at the time of replicating it. In some cases when collecting data, it was not possible to establish whether survival was specifically related to the use of the vasopressor or any other factor. On the other hand, one of the strengths was that, despite being a small sample size, it is a considerable size to have being a single center study. Being a retrospective study, there was no control of the indications for the administered dose of terlipressin. However, despite not being a clinical trial, a follow-up of the dosing protocol was observed according to the recommendations established in literature.

## Conclusion

HRS is a severe complication of cirrhosis, associated with portal hypertension in advanced stages of the disease, being the paracentesis the main trigger of our study. Currently, the use of vasopressor is the therapy of choice. In our study no significant difference was found between terlipressin treatment compared to norepinephrine for complete resolution of HRS. The general mortality at 90 days was high, being higher in patients with HRS1, nonetheless, while the results in this study are suggestive of clinical information for HRS patients in the Colombian population, they should also be interpreted with caution, given the sample size and the type of study. For this reason, further studies are required, probably of a multicenter type, to broaden the sample size and be able to assess different population groups in Colombia. The foregoing, to project ourselves in the future and compare our results globally.

## Abbreviations

HRS: Hepatorenal syndrome
HRS1: Hepatorenal syndrome Type 1
HRS2: Hepatorenal syndrome Type 2
ICA: International Club of Ascites
MELD: Model for End-Stage Liver Disease
NSAIDs: Non-steroidal anti-inflammatory drugs
